# 630. Protective Role of Outer Membrane Vesicles Released by Carbapenemase-Producing Klebsiella pneumoniae Clinical Isolates Against Carbapenems and Cefiderocol

**DOI:** 10.1093/ofid/ofaf695.197

**Published:** 2026-01-11

**Authors:** Ana M González, Carolina López, Valeria Quiroz, Katherine Soto, Christina Schuh, Alejandro J Vila, Lorena Díaz, Jose M Munita

**Affiliations:** Universidad del Desarrollo, SANTIAGO, Region Metropolitana, Chile; Instituto de Biología Molecular y Celular de Rosario, ROSARIO, Santa Fe, Argentina; Universidad del Desarrollo, SANTIAGO, Region Metropolitana, Chile; Universidad del Desarrollo, SANTIAGO, Region Metropolitana, Chile; Universidad del Desarrollo, SANTIAGO, Region Metropolitana, Chile; Instituto de Biología Molecular y Celular de Rosario (IBR), Rosario, Santa Fe, Argentina; Universidad del Desarrollo - Clinica Alemana, Santiago, Region Metropolitana, Chile; Instituto de Ciencias e Innovación en Medicina, Santiago, Region Metropolitana, Chile

## Abstract

**Background:**

Carbapenemase-producing (CP) carbapenem-resistant *Klebsiella pneumoniae* (CRKP) is a major public health threat. Among them, dissemination of metallo-enzymes -such as New Delhi Metallo-β-lactamases (NDM)- is particularly concerning due to the lack of therapeutic options. Outer membrane vesicles (OMVs) are increasingly recognized as vehicles for resistance spread, but their role in carbapenem resistance remains unclear. Cefiderocol (FDC), a novel siderophore-cephalosporin, has shown promising *in vitro* activity against CP-CRKP. However, recent reports suggest its potency against NDM-producing CRKP might be compromised. We explored OMVs’ role as vehicles for carbapenemases export to protect against carbapenem and FDC activity.

**Figure 1.**
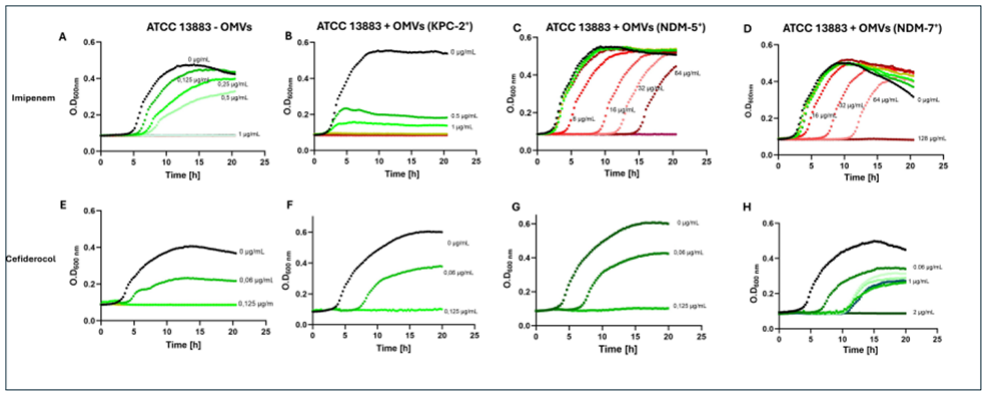
Growth of K. pneumoniae ATCC 13883 co-incubated with OMVs in antibiotic supplemented media. Growth curves of the susceptible strain K. pneumoniae ATCC 13883 in the presence of imipenem (A – D) and cefiderocol (E – H) and co-incubated with OMVs without carbapenemases (A and E) and OMVs loaded with KPC-2 (B and F), NDM-5 (C and G) and NDM-7 (D and H). Green indicates concentrations below the antibiotic breakpoint, red indicates those above.

**Methods:**

OMVs were isolated from three CP-CRKP clinical strains recovered from invasive infections in Chile. Isolates carried different carbapenemase genes: *bla*_NDM-7_, *bla*_NDM-5_, and *bla*_KPC-2_. The presence of NDM and KPC in OMVs was verified by Western blot, and enzymatic activity was assessed with nitrocefin. To assess the protective role of OMVs, antimicrobial-susceptible *K. pneumoniae* ATCC 13883 was co-incubated with purified OMVs and exposed to increasing concentrations of imipenem (IPM) or FDC. Growth kinetics were used to assess antimicrobial activity.

**Results:**

OMVs were successfully purified from all clinical isolates. Western blot revealed high levels of NDM in OMVs, while KPC-2 was weakly detected. Co-incubation of ATCC 13883 with OMVs containing NDM-5 or NDM-7 led to significantly higher growth under IMP concentrations ≥4 µg/mL, and improved growth under increasing FDC concentrations. Interestingly, OMVs with NDM-7 showed growth at higher FDC concentrations than those with NDM-5 (1 vs 0.06 μg/mL). In contrast, KPC-OMVs conferred limited protection, with growth only observed at low IPM concentrations (< 0.5 μg/mL) (Figure 1).

**Conclusion:**

OMVs from NDM-producing CRKP enhanced bacterial survival under IPM and FDC exposure. NDM-7 OMVs conferred stronger FDC protection than NDM-5 OMVs. These findings underscore OMVs’ potential role in antimicrobial resistance beyond genetic transfer. Further research is needed to clarify their contribution.

**Disclosures:**

Alejandro J. Vila, PhD, SHIONOGI and CO., LTD.: Grant/Research Support Jose M. Munita, MD, MSD LATAM: Grant/Research Support|Pfizer, Inc.: Grant/Research Support

